# Review: Influence of the CYP450 Genetic Variation on the Treatment of Psychotic Disorders

**DOI:** 10.3390/jcm10184275

**Published:** 2021-09-21

**Authors:** Lorena Carrascal-Laso, María Isidoro-García, Ignacio Ramos-Gallego, Manuel A. Franco-Martín

**Affiliations:** 1 Servicio de Psiquiatría, Hospital Provincial de Zamora, IBSAL, 49071 Zamora, Spain; lorenacarraslaso@gmail.com (L.C.-L.); mfrancom@saludcastillayleon.es (M.A.F.-M.); 2 Farmacogenética y Medicina de Precisión, Servicio de Bioquímica, Hospital Universitario de Salamanca, IBSAL, 37007 Salamanca, Spain; 3 Departamento de Medicina, Universidad de Salamanca, 37007 Salamanca, Spain; 4 Departamento de Fisiología y Farmacología, Universidad de Salamanca, 37007 Salamanca, Spain; ignramos@usal.es

**Keywords:** schizophrenia, antipsychotics, psychopharmacology, genetics, gene expression

## Abstract

Second-generation antipsychotic metabolism is mainly carried out by the CYP450 superfamily, which is highly polymorphic. Therefore, knowing the influence of the different known CYP450 polymorphisms on antipsychotic plasmatic levels and, consequently, the biological effect could contribute to a deeper knowledge of interindividual antipsychotic treatment variability, prompting possible solutions. Considering this, this state of the art review aimed to summarize the current knowledge about the influence of the diverse characterized phenotypes on the metabolism of the most used second-generation antipsychotics. Forty studies describing different single nucleotide polymorphisms (SNPs) associated with the genes CYP1A2, CYP2D6, CYP3A4, CYP3A5, and ABCB1 and their influence on pharmacokinetics of olanzapine, clozapine, aripiprazole, risperidone, and quetiapine. Most of the authors concluded that although significant differences in the pharmacokinetic parameters between the different phenotypes could be observed, more thorough studies describing pharmacokinetic interactions and environmental conditions, among other variables, are needed to fully comprehend these pharmacogenetic interactions.

## 1. Introduction

Schizophrenic disorders are a group of severe mental diseases that have an estimated annual incidence of 15.2 per 100,000 patients, though the exact prevalence can oscillate between 3.3‰ and 7.2‰ [1]. Schizophrenia is mainly considered a chronic disorder that may follow several patterns that partly define the illness prognosis.

One of the conditioning factors of the prognosis is the response to antipsychotic agents. Antipsychotic drugs are the fundamental piece of the treatment of schizophrenia and other psychotic disorders [2], but for most patients, this therapeutic approach is not totally effective [3]. Interindividual variability in treatment response is thought to be related to diverse factors including ambient, iatrogenic, and genetic factors that can alter pharmacokinetic and/or pharmacodynamic parameters of the drug, or drugs, prescribed. Due to the actual difficulties found when predicting the patient treatment response, the usual course of action of the physician is based on trial-and-error strategies [2], which involve multiple changes of the medication and dose to achieve the most optimal efficiency/security balance possible. This strategy usually results in polypharmacy, which is a method hardly supported by scientific evidence; it does not imply a greater therapeutic effect and is frequently associated with more frequent and severe adverse effects [4]. Furthermore, antipsychotic-based polypharmacy entails a huge economic burden for health services from the healthcare perspective due to a mortality and morbidity rise that lowers the life quality of the patients and from an economic perspective due to an increase in direct and indirect costs [5]. Therefore, instauration of a protocol of rational prescription of antipsychotics is necessary in order to achieve greater optimization in resource allocation.

Most clinical guides related to the treatment of schizophrenia emphasize the need of using monotherapy and avoiding the combination of psychoactive drugs due to the negative influence on the evolution and prognosis of patients [6,7]. However, an overview of clinical practice brings out the wide use, more than 50% of cases, of polypharmacy strategies, that, as said, put at risk patients without enhancing efficacy [8]. Moreover, it implies a higher risk of noncompliance and relapse related to adverse and secondary effects caused by the combination of antipsychotics, such as weight gain, digestive disorders, cardiovascular alterations, and metabolic syndrome [9]. On the other hand, literature reviews show different studies which establish that these interindividual differences imply varying degrees of vulnerability to adverse effects produced by second-generation antipsychotics [10,11].

For the purpose of avoiding the previously described issues, a possible approach is personalized therapy [12,13] adapted to the pharmacogenetic profile of the patient after the determination of biological markers, which could help to predict the antipsychotic tolerability and efficacy [14]. Recently, pharmacogenetics—a scientific discipline that studies the genetic variations involved in the response to drugs—and its potential therapeutic tools have progressed vastly, and their use in a clinical context could help to improve the adjustment of pharmacotherapy to the individual characteristics of the patients; therefore, it turns out to be an essential mainstay for precision medicine [15,16,17,18].

The following antipsychotics included in this study are listed as atypical: amisulpride (oral), aripiprazole (oral/IM depot), asenapine (oral), clozapine (oral), levomepromazine (oral), olanzapine (oral), paliperidone (oral/IM depot), quetiapine (oral), and risperidone (oral/IM depot).

Generally, antipsychotics are metabolized by enzymatic complexes, like the cytochrome P450 system which has the main role in the metabolism and elimination of them, and this could account for its influence on effectivity and toxicity. The CYP450 gene superfamily contains 117 genes grouped in 18 families; CYP1A2, CYP2D6, and the CYP3A subfamily are examples of the genes coding enzymes that are labeled as relevant for antipsychotic drug metabolism. The relationship between the antipsychotics included in this study and the CYP450 enzymatic complex is summarized in Table 1.

Some of the members of this superfamily are highly polymorphic, and this issue presents an important factor when studying the interindividual variability of drug pharmacokinetics as polymorphisms associated with each gene could alter the expression of the said gene or the activity of the coded protein, resulting in different metabolic phenotypes that lead to different plasmatic levels of various drugs and therefore to an altered response to them.

These variations might cause specific patients to poorly metabolize a group of drugs associated with a specific CYP450 enzyme, hereinafter referred to as poor metabolizers, and therefore require lower doses to avoid the emergence of secondary effects. The counterpart of this phenomenon is caused by other genetic variations that give rise to a higher metabolic ratio in patients called ultrarapid metabolizers, which require abnormally elevated doses of drugs to achieve a satisfactory therapeutic effect. These examples are based on the assumption of considering a drug the metabolism whereof results in an inactive metabolite; if we were to consider a prodrug, the examples would have to be reversed.

On the other hand, since antipsychotics are drugs the biological targets whereof are found in the central nervous system (CNS) and that, until recently, with the emergence of depot formulations, were manly orally administered, we should consider the role of the different families of xenobiotic transporters when analyzing pharmacogenetic parameters. It is known that there is a relationship between the ABC transporters (to be precise, ABCB1) and various antipsychotics [19].

The primary objective of this review was to describe the scientific evidence available to date about the influence of the most relevant polymorphisms associated with the principal components involved in the pharmacokinetics of the most used antipsychotic agents. As the secondary objective, the synthesis of the obtained information regarding the clinical application of an extensive pharmacogenetic analysis for the purpose of adjustment of antipsychotic treatment of psychiatric patients, if available, is included.

## 2. Materials and Methods

In order to achieve these objectives, the search was focused on the studies published between January 2009 and July 2021, in which the pharmacokinetic parameters related to the antipsychotic treatment of individuals over 16 years were evaluated by pharmacogenetic testing, within the framework of an empirical investigation, alongside evaluating the psychopathological status of the patients and/or the adverse effects associated with the use of psychoactive drugs and/or the plasma levels of the metabolites derived from the drug or the metabolites associated with a side effect of the drug.

Since one of the primary objectives of this study to evaluate the potential clinical application of pharmacogenetic testing was justified by the influence on pharmacokinetic parameters of different genetic variants, it was not possible to find studies to date that would be randomized clinical trials.

The search was restricted to the scientific articles published, or in the process of being published, in English. In the case of several study populations in the same study, all of them would be reviewed if they had the same size, tracking time and study design. In the event of finding articles published by the same publishing group or by different groups related in some way, the population with the best design would be included in case the population, publication abstract, studied gene or drug, or the information acquired from both articles were coincidental.

The mainly used database was PubMed; the following MeSH Terms were applied: “Antipsychotic Agents” [MeSH], “Schizophrenia Spectrum and Other Psychotic Disorders” [MeSH] “Affective Disorders, Psychotic” [MeSH], “Psychotic Disorders” [MeSH], “Schizophrenia” [MeSH], “Cytochrome P-450 Enzyme System” [MeSH], “Cytochrome P-450 CYP2D6” [MeSH], “Cytochrome P-450 CYP1A2” [MeSH], “Cytochrome P-450 CYP3A” [MeSH], “Risperidone” [MeSH], “Aripiprazole” [MeSH], “Clozapine” [MeSH], “Olanzapine” [MeSH], “Quetiapine Fumarate” [MeSH]; combining each MeSH term referring to an antipsychotic drug with each CYP450 member (et vice versa) and each MeSH term referring to psychotic behaviors using the Boolean Terms “AND” and “OR”.

In the identification process, after disposing of the duplicates, 465 records were included; 455 of them were obtained in the process of systematic search in PubMed and 10 of them were obtained by manual search from publication references. In the screening process, 405 records were eliminated after analysis of the title, abstract and the content extracted by superficial reading.

**Figure 1:**
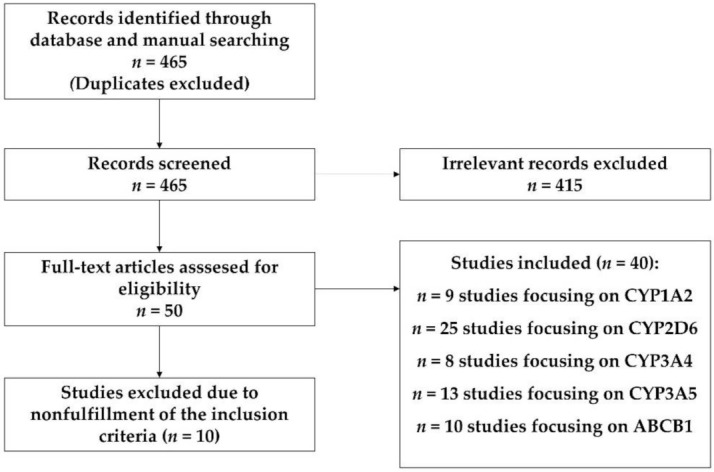
PRISMA pipeline.

In the eligibility process, 48 articles were chosen after deep reading, disposing of 10 articles due to coincidental study populations, incomplete information, or non-fulfilment of the inclusion criteria (PRISMA, [20]) (Figure 1).

## 3. Results

A total of 40 studies were included for data extraction. The characteristics of this studies are summarized in Supplementary Table S1. All the studies but seven [21,22,23,24,25,26,27,28] were performed on psychiatric patients. The effects of the studied genes variability on risperidone metabolism were described in 17 studies; four studies were focused on olanzapine; 12—on aripiprazole; six—on quetiapine, and eight—on clozapine (Supplementary Materials, Table S1).

### 3.1. CYP1A2

CYP1A2 (cytochrome P450 family 1 subfamily A member 2) is a gene located in 15q24.1, that is inducible by polycyclic aromatic hydrocarbons found in smoke (i.e., from cigarettes, fossil fuels, smoked food, etc.) and codes hemotiolate monooxygenase located in the endoplasmic reticulum. To date, this enzyme has no known endogenous substrate. Among the xenobiotic substrates associated with this enzyme are caffeine, aflatoxin B1, acetaminophen, aromatic polycyclic hydrocarbons, etc. Like most members of the CYP450 system, its expression in adults takes place mostly in the liver (see NCBI/Gene) [29].

Among the atypical antipsychotics marketed in Europe, cytochrome CYP1A2 has a major role in the metabolism of olanzapine, clozapine, and asenapine and a minor role in the metabolism of levomepromazine (Table 1) [30]. In the coding region of this cytochrome, there are 41 haplotypes described to date (see PharmVar.org).

CYP1A2*1F (PharmVar; rs762551 (dbSNP accession ID)), contains a −163C > T SNP in intron 1 of the CYP1A2 gene, present in 67.1% of the 125,568 samples recorded in the NCBI SNP database, that is thought to have influence in the inducibility of the gene [31,32,33,34]. The independent studies included in this analysis showed a lower rate of response to treatment with clozapine [35] and significantly lower dose/BMI-corrected olanzapine and clozapine plasma concentrations [36,37] in patients carrying the *1F/*1F genotype compared to subjects with at least one wild-type allele for CYP1A2.

Regarding the influence of the CYP1A2*1F allele on the inducibility of the CYP1A2 gene, the difference in dose/BMI-corrected olanzapine plasma concentrations between the CYP1A2*1F homozygote carriers and the CYP1A2*1A (wild-type allele) homozygote carriers was exposed to be equivalent with or without induction (carbamazepine, smoking) [36], whereas the plasma levels of clozapine were found to be significantly deprecated in *1F/*1F smokers [38,39].

Furthermore, it was shown that carriers of the less inducible allele (wild-type) endured an increase in limb–truncal tardive dyskinesia severity associated with epy neuroleptics metabolized by this cytochrome and were more inclined to suffer tardive dyskinesia [40].

Searching for the possible molecular mechanism that explains the enhanced inducibility of CYP1A2 rs762551 from an in vivo approach, a haplotype constructed by CYP1A2 rs762551, CYP1A1 rs2470893, CYP1A1 rs2472297, CYP1A2 rs2472304, and AHR rs4410790 has been found to be related to a significant increase in the desmethyl olanzapine/olanzapine ratio and a decrease in the olanzapine C/D ratio [41] dependent on the smoking status [42].

CYP1A2*1D (PharmVar; rs35694136 (dbSNP ID)) contains a −2467delT SNP in the 5′ flanking region of CYP1A2 [32], present in 24.5% of the 125,568 samples recorded in the NCBI SNP database, that was found to be associated with higher dose/body weight-corrected olanzapine serum concentrations [37].

CYP1A2*1C (PharmVar: rs2069514 (dbSNP ID)) contains a −3860G > A SNP in the 5′ flanking region of CYP1A2 associated with decreased enzyme activity in vivo [43], present in 13.9% of the 125,568 samples recorded in the NCBI SNP database. Homozygote carriers of the CYP1A2*1C allele showed a higher quetiapine’s AUC than carriers of the *1/*1 or *1F/*1F genotype [26].

More recent studies suggest that CYP1A2 (rs2069514) and ABCB1 (rs1045642, rs1128503, rs2032582, and rs2235048) polymorphisms do not have an influence on the emergence of olanzapine-related adverse effects [44], whereas UGT1A4 polymorphisms have been found to be significantly associated with variability of olanzapine’s pharmacokinetic parameters [41,44]. The rs2470890 (dbSNP ID) SNP (haplotype not determined) contains a 1545 C > T SNP in exon 6 that results in a synonymous variant not reported in ClinVar. CYP1A2 1545 T/T genotype carriers receiving clozapine treatment were found to have a higher average LUNSERS score and a higher need for mood stabilizers administration, although this is not correlated with differences in clozapine exposure [39].

### 3.2. CYP2D6

CYP2D6 (cytochrome P450 family 2 subfamily D member 6) is a gene located in 22q13.2 that codes hemotiolate monooxygenase located in the endoplasmic reticulum. CYP2D6 metabolizes up to 25% of the commonly prescribed drugs, among which antidepressants, antipsychotics, analgesics, antitussives, betablockers, antiarrhythmics, and antiemetics stand out. Like most members of the CYP450 system, its expression in adults takes place mostly in the liver, followed up, to a much lesser extent, by the small intestine, predominantly the duodenum [45].

Among the atypical antipsychotics marketed in Europe, cytochrome CYP2D6 has a major role in the metabolism of aripiprazole and risperidone and a minor role in the metabolism of olanzapine, quetiapine, clozapine, and asenapine (Table 1) [30].

CYP2D6 is a highly polymorphic gene. In the coding region of this gene, there are 129 haplotypes described to date (see PharmVar.org). The usual approach to the relationship between genotypic variability and the metabolism of CYP2D6 substrates is based on the definition of metabolic phenotypes with their characteristic pharmacokinetic implications based on different genetic mechanisms. Poor metabolizers (PM) are associated with two inactive alleles. The combination of two reduced-activity alleles, or a reduced-activity allele with an inactive allele, or an inactive allele with an active allele originates an intermediate metabolizer (IM). An individual with two wt-like alleles is labeled as an extensive metabolizer (EM). The presence of duplication in the absence of inactive or reduced-activity alleles results in an ultrarapid metabolizer (UM) [46].

PM individuals were found to have higher aripiprazole/risperidone active moieties exposure [21,28,47,48,49,50,51], with this exposure even being proportional to the number of affected alleles in some studies [47,48]. Furthermore, it has been found that PM individuals tend to have lower aripiprazole/risperidone doses administered, as it has been recommended in some studies [28,52], and, alongside UM individuals, have a higher risk of risperidone treatment failure [51]. Although several studies tried to define a relationship between poor metabolizers and hyperprolactinemia [53], until now, no statistically significant evidence has been found. Concerning quetiapine metabolism, some studies have suggested that PMs present with higher exposure [54].

Concerning the metabolites linked to the secondary effects, the EM and UM phenotypes in healthy adults are associated with lower prolactin concentrations as compared to the PM and IM phenotypes [55]. The boundaries between phenotypes are yet to be more thoroughly defined. It has been found that, regarding the variability of risperidone pharmacokinetic parameters between CYP2D6-based phenotypes, in healthy individuals, the coefficient of variation for the risperidone AUC and Cmax is higher in IM and EM than in PM and UM [27] and the presence of at least one allele of CYP2D6*10, where, according to the classical phenotypical approach, the combination of this allele with a wt-like one would be labeled as an EM, results in higher mean C/D ratios of aripiprazole and aripiprazole serum concentrations, and the number of CYP2D6*10 alleles significantly affects dose-corrected plasma risperidone levels [53]. Comparing different subtypes of IM, the presence of a combination of a nonfunctional allele (i.e., *3, *4, *5, *6) with a reduced-function allele (i.e., *9, *10, and *41) was found to be associated with higher risperidone/aripiprazole serum concentrations than the serum levels found in wt/nonfunctional carriers. Furthermore, the presence of two copies of reduced-function alleles was found to be associated with higher risperidone/aripiprazole serum concentrations than in the case of the wt/nonfunctional combination, with the levels of nonfunctional/reduced-function carriers being higher than those of the reduced-function/reduced-function carriers [56].

CYP2D6*4 (PharmVar; rs3892097 (dbSNP ID)) contains a 1847G > A SNP, present in 13.8% of the 172,402 samples recorded in the NCBI SNP database, that results in an afunctional enzyme due to a splicing defect/frameshift mechanism. An association between this allele and limb–truncal tardive dyskinesia has been found [40].

### 3.3. CYP3A4

CYP3A4 (cytochrome P450 family 3 subfamily A member 4) is a gene located in 7q22.1 that is inducible by glucocorticoids and some drugs and codes hemotiolate monooxygenase located in the endoplasmic reticulum. CYP3A4 metabolizes up to 50% of the commonly prescribed drugs and is associated with the synthesis of cholesterol, steroids, and other lipid derivates. Like most members of the CYP450 system, its expression in adults takes place mostly in the liver, followed up, to a much lesser extent, by the small intestine, predominantly the duodenum [57].

Among the atypical antipsychotics marketed in Europe, cytochrome CYP3A4 has a major role in the metabolism of aripiprazole, clozapine, quetiapine, and levomepromazine and a minor role in the metabolism of risperidone [30].

CYP3A4*1G (PharmVar; rs2242480 (dbSNP ID)) contains a 20230G > A SNP in exon 10 of the CYP3A4 gene, not yet fully enzymologically characterized [58], present in 22.29% of the 245,260 samples recorded in the NCBI SNP database, that has been associated with a lower PANSS score in risperidone-treated drug-naive schizophrenic patients [59].

CYP3A4*22 (PharmVar; rs35599367 (dbSNP ID)) contains a 15389C > T SNP in intron 6, associated with decreased enzyme expression in vivo [60,61,62], present in 3.2% of the 125,568 samples recorded in the NCBI SNP database. In various retrospective studies, CYP3A4*22 was found to be associated with higher quetiapine and risperidone plasma levels compared to the wild-type allele [49,63]. The role of the CYP3A4*22 allele in the metabolism of aripiprazole has been evaluated in some studies [28] and a relationship between this allele and slightly increased serum levels has been found [49]. It has been suggested that CYP2D6 could take the aripiprazole-metabolizing role of CYP3A4 in patients with the latter altered; that could explain the absence of a bigger effect of CYP3A4*22 [64].

One study suggested that the influence of CYP3A4 variants on aripiprazole metabolism is neglectable as the clearance of this drug is mainly carried out by CYP2D6, and consequently the presence of CYP2D6 non-wt genotypes and inhibitors being responsible for the interindividual variability. The apparent relationship between a reduced CYP3A4 activity and an inefficient aripiprazole therapy could be the consequence of an unnoticed phenocopy effect [65].

### 3.4. CYP3A5

CYP3A5 (cytochrome P450 family 3 subfamily A member 5) is a gene located in 7q22.1 that codes a monooxygenase involved in drug metabolism and synthesis of cholesterol, steroids, and other lipids. Its expression in adults takes place mostly in the gastrointestinal tract, mainly the small intestine (duodenum) and the stomach [66].

CYP3A5*3 (PharmVar; rs776746 (dbSNP ID)) contains a 6986A > G SNP located in exon 3 of CYP3A5 that results in a splicing defect associated with decreased enzyme activity in vivo [67,68,69,70], present in 71.08% of the 125,568 samples recorded in the NCBI SNP database. CYP3A5*3 has been found to reduce the dehydroaripiprazole/aripiprazole ratio in homozygous carriers in a much weaker way than the presence of CYP2D6 PMs [28] and associated with higher plasma levels of quetiapine on homozygous and heterozygous carriers [24]. Some authors conclude that the effect of this SNP in the pharmacokinetics of aripiprazole [71] and quetiapine is minimal but should be studied in patients with possible drug–drug interactions [25,54].

### 3.5. ABCB1

ABCB1 (ATP-binding cassette subfamily B member 1) is a gene located in 7q21.12 that is member of the MDR/TAP subfamily that is part of the ABC transporter superfamily. It codes a membrane ATP-dependent efflux bomb that carries xenobiotics and displays a high number of substrates. Its expression in adults takes place mostly in the adrenal gland, the intestines, the liver, the kidneys, and the brain. There are various SNPs described in the coding region of this gene registered in the NCBI dbSNP database (1159 cSNPs) [72].

The rs1045642 variant contains a 3435C > T synonym SNP located in exon 26 of the ABCB1 gene, present in 50.2% of the 246,062 samples recorded in the NCBI SNP database, associated with a reduced expression of the gene [73]. A relationship between the altered allele and an increase in the exposure to risperidone active moieties has been found [71].

The rs1128503 variant, present in 53.5% of the 246,028 samples recorded in the NCBI SNP database, contains a 1236T > C synonym SNP located in exon 8 of the ABCB1 gene [74]. It has been found that patients carrying this SNP present with lower clearance of aripiprazole [28].

The rs2032582 variant, present in 57.4% of the 245,398 samples recorded in the NCBI SNP database, contains a 2677T > G missense SNP located in exon 16 of the ABCB1 gene [74]. Although several studies focused on the relationship between the ABCB1 rs2032582–rs1128503–rs1045642 haplotype and quetiapine [24,25], no significant differences were found in the pharmacokinetic parameters between the altered and wt carriers.

### 3.6. Summary

Regarding the extracted data about the relationship between the genetic variability related to cytochrome CYP1A2 and the atypical antipsychotics metabolized by themselves, eight studies were found, four of which were focused on the metabolism of olanzapine, three—on the metabolism of clozapine. All the studies discovered some influence of the studied polymorphisms on the studied drug plasma levels with strong statistical significance except for one, with some controversy when defining the role of CYP1A2 in the metabolism of olanzapine (Table 2).

With respect to the extracted data about the relationship between the genetic variability related to cytochrome CYP2D6 and the atypical antipsychotics metabolized by themselves, twenty-four studies were found, thirteen of which were focused on risperidone metabolism, six—on aripiprazole metabolism, two—on quetiapine metabolism, and one—on the metabolism of olanzapine. In twelve of these studies, a statistically significative relationship was found between the aripiprazole/risperidone plasma levels and the PM phenotype. One article was focused on the variability of risperidone metabolism between individuals labeled with the same phenotype while carrying diverse genotypes and exposed that in the nonextreme phenotypes (EM and IM), variability between individuals was less pronounced than among the extreme phenotypes. Regarding aripiprazole and risperidone metabolism, differences were also found between the different genotypes that make up the IM phenotype. Furthermore, an allele of reduced activity (* 10) correlated with higher levels of quetiapine in plasma (Table 2).

Concerning the extracted data about the relationship between the genetic variability related to cytochrome CYP3A4 and atypical antipsychotics metabolism, eight studies were found, three of which were focused on the metabolism of risperidone, two—on aripiprazole, two—on quetiapine, one—on olanzapine, and one—on the metabolism of clozapine. Although a relationship between CYP3A4*22 and the variability in aripiprazole metabolism was described, it was not statistically significant, requiring a more thorough analysis with better control of variables. A slight correlation was found between the CYP3A4*1G haplotype and a defect in risperidone metabolism

With respect to the extracted data about the relationship between the genetic variability of cytochrome CYP3A5 and the atypical antipsychotics metabolized by it, ten studies were found, three of which were focused on the metabolism of risperidone, two—on aripiprazole, four—on quetiapine, and one—on the metabolism of olanzapine. As described in these studies, there is a very weak correlation between polymorphisms in this gene and the plasma levels of the studied drugs, which is supported by the fact that this enzyme has a limited role in antipsychotic metabolism, and its study is possibly interesting in cases of drug interactions or depending on the patient’s pharmacogenetic status and environmental conditions. All these data should be considered when applying personalized treatment fitted to the metabolism of the patient, potentially reducing side effects frequency and severity and improving the efficacy of the treatment.

Regarding the data extracted on the genetic variability of cytochrome ABCB1 in relation to the antipsychotics metabolized by it, eleven studies were found; five of them focused on the metabolism of risperidone, two—on the metabolism of aripiprazole, one—on the metabolism of quetiapine, and two—on clozapine metabolism. Although some studies have found correlation of some polymorphisms with the variation in the clearance of aripiprazole and risperidone, there is no strong statistical significance in most studies focused on this gene.

## 4. Discussion

The CYP450 members 2C8, 2C9, and 2C19 were not included in this analysis because of the scarcity of bibliographical material about the evidence of its clinical influence in antipsychotic-treated patients. In this review, we focused our search on the four genes associated with antipsychotic metabolism and one known to be related to the blood–brain barrier and enterocytic transport of antipsychotics.

Based on the conclusions of the most recent studies, it seems that CYP1A2 polymorphisms only influence CYP1A2-metabolized antipsychotic drugs by altering their inducibility, making smokers carrying CYP1A2 *1F especially vulnerable to possible pharmacokinetic conflicts. Some reviewers consider that CYP1A2 does not have a sufficiently notable impact on olanzapine and clozapine [75,76], whereas others explain the controversy regarding the influence of the CYP1A2 genetic variability on clozapine plasma levels [77]. From our point of view, CYP1A2 genetics and pharmacokinetics related to antipsychotic drugs needs to be more thoroughly studied to reach more definite conclusions but CYP1A2 polymorphisms could be clinically relevant when considering patients exposed to inductors (such as smoke), and the prescriber should be particularly aware when planning the pharmacotherapy of such patients.

Some reviewers have already analyzed the evidence for the impact of CYP2D6 genetic polymorphisms and risperidone pharmacokinetics, concluding that a reduced function of this enzyme is associated with increased exposure to its active moieties, particularly in PMs [78,79]. At the same time, CYP2D6 is the most extensively studied CYP450 enzyme and therefore, we are aware of the large variety of possible genotypes present even only considering the Caucasian population. With our current knowledge of CYP2D6-metabolized antipsychotic drug pharmacokinetics, it appears that CYP2D6 “loss-of-function” and “reduced-function” polymorphisms should be considered clinically relevant when considering patients administered with risperidone or aripiprazole, in particular PMs, but the IMs’ phenotypical variability is still a controversial matter. Clinicians should be specifically aware of possible phenoconversions in IM-predicted patients due to pharmacokinetic drug–drug interactions.

Due to the scarcity of literature describing the role of CYP3A4 in the metabolism of antipsychotics with an in vivo approach, it is difficult to reach conclusions about the potential relevance of CYP3A4 testing in a clinical setting. Further studies describing this matter that also consider possible phenocopy effects and different factors influencing pharmacokinetics of CYP3A4-metabolized antipsychotics are needed.

The influence of the CYP3A5 genetic variability on antipsychotic metabolism is difficult to ascertain as it is considered to be a minor metabolic pathway of some of the antipsychotic drugs included in this study. As patients diagnosed with a psychotic syndrome tend to be prescribed with multiple drugs, it is considered that CYP3A5 could take a more relevant role in the metabolism of antipsychotic drugs when drug–drug pharmacokinetic interactions occur or when the other possible metabolic pathways are diminished due to a polymorphism.

The relevance of ABCB1 polymorphisms when considering the interindividual variability of antipsychotic treatment response is still a matter of controversy. This correlates with the conclusions of previous reviews [76]. An in vitro approach to further define the influence of the most known polymorphisms of ABC1 on the transportation kinetics of antipsychotics through this pathway could be helpful to design future in vivo studies.

One of the main complications found in this type of studies is the phenocopy effect. Patients with similar genotypes are sometimes considered equivalent from the pharmacokinetic perspective, without analyzing dietary and toxic habits, concomitant pharmacotherapy, age, or sex. Analysis of the pharmacogenetic testing results should be performed with a deep understanding of the patient’s pharmacokinetic context and being aware of the possible drug–drug or diet–drug interactions. Furthermore, studies focusing on various genes covering the whole pharmacokinetic pathway of the drug or drugs prescribed to the patient could increase our knowledge about the possible secondary pathways and the role of secondary metabolizers.

## 5. Conclusions

Most authors agree on the need for more thorough studies emphasizing variables such as the patient’s pharmacokinetic interactions, the pharmacogenetic status of the rest of the CYP450 system, and the patient’s environmental conditions. The purpose of this study was to review and update the usefulness of pharmacogenetics for clinical psychiatry in order to provide help for more personalized prescriptions. The use of pharmacogenetics will help psychiatry to find better outcomes, and it should be used in the regular clinical activity.

The mentioned phenocopy effect could be responsible for the controversy regarding the role of different CYP450 enzymes in the metabolism of antipsychotics, with some studies reaching opposite conclusions. Clinicians should take into consideration the patient’s genotype when prescribing antipsychotics while being aware that the metabolic phenotype is influenced by various factors.

## Figures and Tables

**Table 1:** Antipsychotic metabolism summary.

	Principal Metabolizer	Secondary Metabolizer	Minor Metabolizer	Product
Olanzapine ^¶^	CYP1A2	CYP2C8	CYP2D6 *^,†^	Inactive metabolite
Aripiprazole	CYP2D6 ^†^^,§^, CYP3A4		CYP3A5	Active metabolite
Risperidone	CYP2D6 ^†^		CYP3A4 ^†^	Active metabolite (paliperidone)
Amisulpride	NO CYP			
Clozapine	CYP1A2 ^†^, CYP3A4 ^†^^,§^	CYP2C19 ^†^, CYP2C9 ^†^, CYP2D6 ^†^	CYP3A5 ^†^	Active metabolite, inactive metabolite (CYP1A2/CYP3A4)
Paliperidone	NO CYP			
Quetiapine	CYP3A4		CYP3A5, CYP2D6 ^*,^^†^	Inactive metabolite
Asenapine	CYP1A2	CYP2D6 ^‡^		Inactive metabolite
Levomepromazine	CYP3A4 ^†^	CYP1A2		Inactive metabolite

* Minimal influence on plasma levels, ^†^ substrate inhibition, ^‡^ suicide substrate, ^§^ inductor, ^¶^ CYP3A4 inhibition.

**Table 2:** Summary of the results.

Reference	SNP(s)	Antipsychotic	Influence on Pharmacokinetic Parameters
Laika et al., 2009	CYP1A2: rs762551	Olanzapine	↓PL
Söderberg et al., 2013	CYP1A2: rs762551, rs2472304/AHR: rs4410790, rs4410790/CYP1A1: rs2470893, rs2472297	Olanzapine	↓C/D ↑M/D
Czerwesnky et al., 2015	CYP1A2: rs762551, rs35694136	Olanzapine	rs762551: ↓PL; rs35694136: ↑PL
Vikki et al., 2014	CYP1A2: rs2470890	Clozapine	↓PL
Balibey et al., 2011	CYP1A2: rs762551	Clozapine	↓RR
Huang et al., 2016	CYP1A2: rs762551	Clozapine	↓PL
Cabaleiro et al., 2015	CYP1A2: rs2069514	Quetiapine	↑AUC
Yan et al., 2020	CYP1A2: rs2069514	Olanzapine	None
Hattori et al., 2020	CYP1A2: rs2069514	Olanzapine	↓C/D
Novalbos et al., 2010	CYP2D6 (PM)	Risperidone	↑PL
Nagai et al., 2013		Aripiprazole	↑PL
Suzuki et al., 2014		Ariprazole	↑PL
Bakken et al., 2015		Quetiapine	↑PL
Van Der Weide et al., 2015		Ariprazole/risperidone	↓AD
Lisbeth et al., 2015		Risperidone	↑PL
Belmonte et al., 2017		Aripiprazole	↑PL/↓AD
Jukic et al., 2019		Aripiprazole	↑PL
Koller et al., 2020	CYP2D6	Aripiprazole	↑Prolactine (PL, AUC, C_max_)
Du et al., 2009	CYP3A4: rs2242480	Risperidone	↓PANSS
Van der Weide et al., 2014	CYP3A4: rs35599367	Quetiapine	↑PL
Van der Weide et al., 2015	CYP3A4: rs35599367	Aripiprazole/risperidone	↑PL
Belmonte et al., 2015	CYP3A5: rs776746	Aripiprazole	↓ M/D
Kim et al., 2014	CYP3A5: rs776746	Quetiapine	↑PL
Suzuki et al., 2014	ABCB1: rs1045642	Risperidone	↑PL
Belmonte et al., 2017	ABCB1: rs1128503	Aripiprazole	↑PL

**RR: response rate.** PL: plasma levels. C/D: concentration/dose ratio. M/D: metabolite/drug ratio. AD: administered dose. PANSS: positive and negative syndrome scale. AUC: area under the curve. SNP: single nucleotide polymorphism.

## Supplementary Materials

The following are available online at https://www.mdpi.com/article/10.3390/jcm10184275/s1, Table S1: Study Design Summary.
