# Supplementary material for: Review: Influence of the CYP450 Genetic Variation on the Treatment of Psychotic Disorders
Source: J Clin Med. 2021 Sep 21;10(18):4275. doi: 10.3390/jcm10184275 (PMC8464829; doi:10.3390/jcm10184275)
Supplement: Supplementary file 1 [file jcm-10-04275-s001.zip › jcm-1329293-supplementary.pdf]

Table S1: Study Design Summary

Ref.	*n*	Health Status	Gene(s)	SNP(s)	Gender Distribution	Age (y)	Smokers	Race	Antipsychotic	Daily Dose (mg/d)	Biometric Measures	Plasma Levels	Adverse Effects Tests/ Psycopathologic Status Test	DNA Analysis
Laika et al., 2009	73	Psychotic Disorder/ Mood Disorder/ Other Psychiatric Disorders	CYP1A2	1A2: rs762551	37F/36M	41.7 +- 14.7 (19-76)	30	Caucassian	Olanzapine	2.5-30 mg/d	BMI	LC/ MS-MS	DOTES/PDS/CGI1/ CGI2	RFLP
Kang et al., 2009	64	Schizophrenia	CYP2D6/ CYP3A5	CYP2D6: rs1065852, whole-gene deletion/CYP3A4: rs776746	23F/41M	48.9 +- 3.9	-	Asian (Korean)	Risperidone	5.3 (2 - 16) mg/d	-	HPLC/ ECD	-	allele specific PCR/ ld-PCR/ RFLP
Du et al., 2009	130	Schizophrenia (Drug Naive)	CYP3A4	CYP3A4: rs4986907, rs2242480	85F/45M	36.07 +- 11.22	-	Asian (Han Chinese)	Risperidone	2 - 6 mg/d	-	HPLC/ UV	PANSS	allele specific PCR
Consoli et al., 2009	60	Psychotic Disorder	ABCB1	ABCB1: rs1045642, rs2032582, rs1128503	20F/40M	45.3 +- 15.3	-	Caucassian (Mediterranean)	Clozapine	165 +- 111 mg/d	Body Weight	HPLC/ UV	BPRSt0/BPRSt1	RFLP

Novalbos et al., 2010	71	Healthy	CYP2D6	CYP2D6: rs35742686, rs3892097, rs5030655, rs5030867, rs5030656, whole-gene deletion	36F/35M	23.6 +- 2.1F/23.2 +- 2.7M	-	Cacuassian (Mediterranean)	Risperidone	1 mg*	BMI	HPLC/MS-MS	-	RFLP/ld-PCR/rtPCR
Jovanovic et al, 2010	83	Schizophrenia/Drug Naive	CYP2D6/ABCB1	CYP2D6: rs35742686, rs35742686, rs3892097, rs5030655, duplication, whole-gene deletion/ABCB1: rs2032582, rs1045642	66F/17M	30.29 +- 8.1	45	Caucassian (Croatian)	Risperidone	2.74 +- 1.6 mg/d	Body Weight	HPLC/UV	PANSS/EPS, Amenorrhea, Galactorrhea, Gynecomastia	RFLP/ld-PCR/rt-PCR
Xiang et al., 2010	23	Healthy	CYP2D6/CYP3A5/ABCB1	*4, *5, *10, *14A/*3/C1236T, C3435T	23M	36.5 +- 2.70 (31 - 39)	-	Asian (Han Chinese)	Risperidone	2 mg*	BP/BMI	HPLC/MS-MS	-	RFLP
Balibey et al., 2011	107	Chronic Schizophrenia	1A2	rs762551	16F/81M	18-60	58	Turk	Clozapine	200-600 mg/d	-	-	BPRS/SANS/SAPS	PCR
Suzuki et al., 2011	63	Schizophrenia	2D6	rs1065852	32F/31M	39.4 +- 15.1	16	Asian (Japanese)	Aripiprazole	12 (*n* = 23)/24 (*n* = 40) mg/d	Body weight	LC/MS-MS	-	rt-PCR
Bigos et al., 2011	235	Schizophrenia	CYP1A2/CYP2D6/FMO3/CYP3A4, 3A5, 3A7, 3A43	rs2472300, rs2069522, rs2069526, rs4646425, rs11631682/rs6002616, rs9306356/rs2213712, rs2859228, rs2859229, rs1492899, rs929087, rs10458360/rs2527894, rs2527887, rs4215, rs2525557, rs6960542,	63F/172M	-	150	Caucassian (*n* =161)/African American (*n* = 74)	Olanzapine	7.5 - 30 mg/d	-	HPLC/MS-MS	PANSS	SNP Mass Array

				rs4729562, rs651430, rs12535293, rs472660, rs17161981, rs17161983, rs2572023, rs2527927										
Suzuki et al., 2012	64	Schizophrenia (*n* = 61), Unspecified Psychotic Disorder (*n* = 2), Delusional Disorder (*n* = 1)	CYP2D6	*10, whole-gene deletion	31F/33M	35.5 +- 14.6 (18-65)	25	Asian(Japanese)	Risperidone	4.6 +- 2.4 mg/d	BMI	HPLC/MS-MS	-	rt-PCR/ld-PCR
Nagai et al., 2012	70	Schizophrenia	CYP2D6	CYP2D6: *5 (whole-gene deletion), rs1065852, rs5030865.	34F/36M	38.2 +- 14.1F/39.2 +- 16.4M y	x	Asian (Japanese)	Aripiprazole	12 (*n* = 25), 24 (*n* =46) mg/d	Bodyweight	-	Prolactin Plasma Levels (Chemiluminescene immunoassay)	PCR
Yoo et al., 2012	80	Healthy	CYP2D6/ABCB1	CYP2D6: rs1135840, rs3892097, *5 (whole-gene deletion), rs1065852, rs5030865, rs1058164, rs1135840. ABCB1: rs1045642.	80M	23.4 +- 2.1 (19-30) y	-	Asian (Korena)	Risperidone	2 mg/d	BSA	HPLC/UV	-	PCR
Lee et al., 2012	96	Schizophrenia	ABCB1/HRH1/DRD2-ANKK1	rs7787082, rs10248420/rs13064530/rs4938013	36F/60M	32.5 +- 9.3	-	Asian (Korean)	Clozapine	319.0 +- 133.1 mg/d	-	HPLC/MS-MS	CGI-I	PCR-MALDI TOF/Mass

														Array
Söderberg et al., 2013	342	Psychiatric Patient	CYP1A2/AHR	CYP1A2: rs762551, rs2472304/AHR: rs4410790, rs4410790/CYP1A1: rs2470893, rs2472297.	161F/180M (1 Unknown)	45 (16-89)	85F/109M	Caucassian	Olanzapine	10	-	UPLC/MS-MS	-	SNP&SEQ
Almoguera et al., 2013	75	Schizophrenia	CYP2D6	CYP2D6: rs35742686, rs3892097, rs5030655. CYP3A4: rs2740574, rs4646437, rs35599367. CYP3A5: rs776746. CYP3A7: rs45446698. ABCB1: rs2032582, rs9282564, rs2229109, rs1045642, rs1128503.	34F/41M	>40 (*n* = 25), 40-59 (*n* = 39), >= 60 (*n* = 11)	-	Caucasian (Mediterranean)	Risperidone	3.5 - > 9 mg/d	BMI	x	PANSS (P/N/T), Length of Hospital Stay	rt-PCR
Herbild et al., 2013	207	Pyschiatric Patient	CYP2C19/CYP2D6	CYP2C19: rs4244285, rs57081121. CYP2D6: rs35742686, rs3892097, *5(whole-gene deletion).	92F/115M	41.5 (19-73) y	-	Caucasian (Nordic-Scandinavian)	Various	-	-	-	Health Care Costs	rt-PCR
Hendset et al., 2013	414	Psychiatric Patient	2D6	rs35742686, rs3892097, rs5030655, rs1065852, rs28371725, whole gene deletion	-	37 (8 - 89)/33 (10 - 86)	-	Caucassian	Risperidone/Aripiprazole	3 (0.5 - 10)/15 (2.5 - 45) mg/d	-	UHPLC/MS-MS	-	rt-PCR
Suzuki et al., 2013	74	Schizophrenia	ABCB1	ABCB: rs1045642, rs2032582. CYP2D6: *5 (whole-gene deletion), rs1065852.	36F/38M	37.5 +- 15.1 (18 - 65) y	28	Asian (Japanese)	Risperidone	4.7 +- 2.4 (mg/d)	Body Weight	HPLC/MS	BPRS (28.3 +- 9.0)	PCR
Vikki et al., 2014	187	Schizophrenia/Schizophreniform disorder/Schizoaffective	CYP1A2	CYP1A2: rs2470890.	70F/110M	>18 (43. 1 +- 11.0)	94T	Caucasian (Finnish)	Clozapine	-	-	HPLC/UVD	LUNSERS	TaqMan SNP Genotyping

		disorder/ Delusional disorder												Assay
Van der Weide et al., 2014	238	Psychotic Disorder	CYP3A4	CYP3A4: rs35599367.	150F/88M	71 (18 - 35 y)/100 (36 - 54 y)/67 (>55 y)	x	x	Quetiapine	300 (12.5 - 1200) mg/d	-	HPLC/UV	Quetiapine	rt-PCR
Suzuki et al., 2014	89	Schizophrenia	CYP2D6/ CYP3A5/ ABCB1	CYP2D6: *5 (whole-gene deletion), rs1065852, rs5030865. CYP3A5: rs776746. ABCB1: rs1045642, rs2032582.	43F/46M	37.94 +- 15.16 y	10	Asian (Japanese)	Aripiprazole	12 (*n* = 33), 24 (*n* =56) mg/d	BMI	HPLC/MS	-	PCR
Kim et al., 2014	40	Healthy	CYP3A5/ ABCB1	ABCB1: rs1128503, rs2032582, rs1045642. CYP3A5: rs776746.	40M	23.8 +- 2.6 (19-33) y	-	Asian (Korean)	Quetiapine	100mg/d	BMI (Height, Weight)	HPLC/MS-MS		Pyrosequencing
Czerwesnky et al., 2015	70	Psychotic Disorder	CYP1A2	CYP1A2: rs762551, rs35694136.	54F/44M	41.5 +- 14.6	40	Caucasian	Olanzapine	14.7 +- 7.3 mg/d	BMI	HPLC/MS-MS	-	PCR
Ivanova et al., 2015	319	Schizophrenia/Schizotypical disorder	CYP1A2	CYP1A2: rs762551.	123F/196M	43 +- 0.9	-	Caucassian	Various*	733.6 +- 40 (mg/day, Clorpromazine)	-	-	AIMS (TD)	rt-PCR
Caballeiro et al., 2015	70	Healthy	CYP2D6	rs35742686, rs1135824, rs3892097, rs5030655, rs5030867, rs5030656, rs1065852, rs28371725, rs16947, rs1135840,	35F/35M	28.2 +- 2.6 (25-39)	-	Caucassian (Mediterranean)	Risperidone	1 mg/d	-	HPLC/MS-MS	-	DNA Array

				duplications, whole-gene deletion										
Lisbeth et al., 2015	82	Psychiatric Patient	2D6	rs1135840, rs773790593, rs5030656, rs1065852, rs28371706, rs16947, rs61736512, rs1058164, rs59421388, rs1135840, rs28371725, rs35742686, rs3892097, rs5030655, rs5030867, rs5030865, rs5030863, whole-gene deletion	30F/52M	19 - 66	-	Caucassian	Aripiprazole/Haloperidol/Paliperidone/Risperidone/Zuclopenthixol	¿?	-	UHPLC/MS-MS	-	CYP 2D6 genotyping commercial kit
Van der Weide et al., 2015	834	Psychotic Disorder	CYP2D6/CYP3A4	CYP2D6: rs35742686, rs3892097, *5 (whole-gene deletion), rs5030655, rs5030656, rs1065852, rs28371725. CYP3A4: rs2740574.	480F/354M	18 - 35 (*n* = 233), 36 - 54 (*n* = 252), >= 55 (*n* = 349)	x	Caucasian (Nordic)	Aripiprazole (*n* = 130), Haloperidol (*n* = 312), Pimozide (*n* = 86), Risperidone (*n* = 396)	-	-	HPLC/UV	-	PCR
Brandl et al., 2015	152	Schizophrenia/Schizoaffective disorder	CYP3A4	CYP3A4: rs680055, rs472660.	55F (37/18) /97M (49/48) *	34.9 ± 10.6 (35.3 ± 12.0/34.5 ± 8.5)	x	Caucasians	Various (*n* = *86)*/Clozapine (n = 66)*		-	x	PANSS/BPRS	PCR
Vandenberghe	150	Pyschiatric Patient	CYP2D6/CYP3A4/CYP3A5/		68F/82M	39	x	Caucasian (122), Asian (4), Arab	Risperidone	<2 (*n* = 68), 2.1-4 (*n*	BMI	HPLC/MS	PANSS/SAS	rt-PCR

et al., 2015			CYP3A7/ ABCB1					(3), African (6), Other (15)		= 65), 4.1-6 (*n* = 13), >6 (*n* = 4)				
Shilbayeh et al., 2015	34	Healthy	CYP3A5	CYP3A5: rs776746.	34M	36 (19-46) y	23	Caucasian (North-American)	Quetiapine	25 mg/d	BMI	HPLC/ MS-MS	-	PCR
Cabaleiro et al., 2015	79	Healthy	CYP1A1/ CYP1A2/ CYP2C19/ CYP2C9/ CYP2D6/ CYP3A4/ CYP3A5	CYP1A2: rs2069514, rs762551. CYP2C19: rs4244285, rs28399504. CYP2C9: rs1799853, rs1057910. CYP2D6: rs1135840, rs5030862, rs28371706, rs61736512, *40 [1863_1864ins(TTT CGC CCC)2], rs28371725, rs28371725, rs1058164, rs16947. CYP3A4: rs2740574, CYP3A5: rs776746, rs10264272.	31F/48M	23.80 +- 3.22F/23.40 +- 3.33M	-	Caucasian (Mediterranean)/Guatemalan (*n* = 2)	Quetiapine	-	BMI	-	Prolactin Plasma Levels, Adverse Efects Questioning	PCR
Bakken et al., 2015	289	Psychotic Disorder	CYP2D6/ CYP3A5/ ABCB1	CYP2D6: rs35742686, rs3892097, *5 (whole-gene deletion), rs5030655. CYP3A5: rs28365083, rs776746. ABCB1: rs1045642.	161F/128M	38 (15-82) y	x	Caucasian (Central European)	Quetiapine	600 (25-2000) mg/d	-	HPLC/ MS	-	PCR
Huang et al., 2016	143	Schizophrenia	CYP1A2	CYP1A2: rs762551.	56F/87M	25-83	10F/55M	Han Chinese	Clozapine	-	BMI	HPLC/ MS-MS	-	RFLP

Xu et al., 2016	304	Schizophrenia	COMT/AKT1/CYP2C19	rs737865/rs2494738/rs4986893	125F/179M	39.9 +- 16.4/37.6 +- 14.5	-	Asian (Han Chinese)	Risperidone	2.1 +- 1.2/2.3 +- 1.6 mg/d	-	-	PANSS	PCR
Xu et al., 2016	240	Schizophrenia	ABCB1/HTR2C/DRD3/TNIK/HTR2C/COMT/COMT/TCF4/SLC6A2/SLC6A2/AKT1	rs2032582, rs498177, rs167771, rs2088885, rs3813929, rs4646316, rs4680, rs9960767, rs2242480, rs5569, rs3001371	109F/131M	42.1 +- 12.9/41.7 +- 12.5	-	Asian (Han Chinese)	Clozapine	122.0 +- 69.2/122.2 +- 69.2 mg/d	-	-	PANSS	PCR
Xu et al., 2016	310	Schizophrenia	NOTCH4/DRD3/CYP2D6/COMT/COMT/COMT/COMT/HTR2C	rs3131296, rs6280, rs1135840, rs6269, rs5993883, rs4818, rs1544325, rs1414334	179F/131M	43.51 +- 17.8/43.4 +- 18.4	-	Asian (Han Chinese)	Quetiapine	246.62 +- 130.9/245.9 +- 130.2 mg/d	-	-	PANSS	PCR
Xu et al., 2016	141	Schizophrenia	RELN/DRD3/AKT1/COMT/COMT/CYP2C19	rs7341475, rs6280, rs2494732, rs6269, rs174696, rs4986893	61F/80M	38.4 +- 15.1/37.0 +- 12.7	-	Asian (Han Chinese)	Chlorpromazine	139.7 +- 48.6/144.2 +- 62.3 mg/d	-	-	PANSS	PCR
Kaur et al., 2017	419	Schizophrenia/Healthy	CYP2D6	CYP2D6: rs3892097, rs106585.	157F/262M	31.36	x	Asian (Indian)	Risperidone	-	BMI, PR, BP, WC	HPLC-UV	PANSS (P/N/T)	RFLP
Belmonte et al., 2017	148	Healthy	CYP2D6/CYP3A4/CYP3A5/ABCB1	CYP2D6: rs35742686, rs3892097, *5 (whole-gene deletion), rs5030655, rs5030656. CYP3A4: rs67666821, rs2740574. CYP3A5: rs776746. ABCB1:	63F/85M	18 - 55	x	Caucasian (Mediterranean)	Aripiprazole	10 (mg/d)	-	HPLC-MS/MS	-	PCR

				rs1045642, rs1128503, rs2032582.										
Papazisis et al., 2018	104	Schizophrenia/Other Psychotic Disorders	CYP2D6/ABCB1	CYP2D6: rs35742686, rs3892097, *5 (whole-gene deletion), rs1065852. ABCB1: rs2032582, rs1045642.	31F/70M	42.6 +- 9.64	-	Caucasian (Mediterranean)	Various	943.1 +- 495.25	-	HPLC/MS-MS	PANSS	rt-PCR
Jukic et al., 2019	725	-	CYP2D6	rs35742686, rs3892097, rs5030655, rs5030656, rs1065852, rs28371725, duplication, whole-gene deletion	372F/353M	42.8 +- 20.4	-	Caucassian	Risperidone	2.87 +- 1.65	-	HPLC/MS-MS	-	rt-PCR
Jukic et al., 2019	890	-	CYP2D6	rs35742686, rs3892097, rs5030655, rs5030656, rs1065852, rs28371725, duplication, whole-gene deletion	490F/400M	37.8 +- 16.8	-	Caucassian	Aripiprazole	14.45 +- 7.71	-	HPLC/MS-MS	-	rt-PCR

HPLC: High-Performance Liquid Chromatography ; MS: Mass Spectometry ; rt-PCR: real time-Polymerase Chain Reaction; PANSS: Positive and Negative Syndrome Scale; UV: UltraViolet (spectrophotometry); RFLP: Restriction Fragment Length Polymorphism ; LUNSERS: Liverpool University Neuroleptic Side Effect Rating Scale. ; TOF: Time of Flight ; BMI: Body Mass Index; CGI: Clinical Global Impression; AIMS (TD): Abnormal Involuntary Movement Scale (tardive Dyskinesia); DOTES: Dosage Record Treatment Emergent Symptom Scale; PDS: Post-Traumatic stress Diagnostic Scale ; BPRS: Brief Psychiatric Rating Scale; SANS: Scale for the Assessment of Negative Symptoms; SAPS: Scale for the Assessment of Positive Symptoms.
